# Kinase and channel activity of TRPM6 are co-ordinated by a dimerization motif and pocket interaction

**DOI:** 10.1042/BJ20131639

**Published:** 2014-05-13

**Authors:** Jenny vanderWijst, Maxime G. Blanchard, Helen I. Woodroof, Thomas J. Macartney, Robert Gourlay, Joost G. Hoenderop, René J. Bindels, Dario R. Alessi

**Affiliations:** *MRC Protein Phosphorylation and Ubiquitylation Unit, College of Life Sciences, University of Dundee, Dow Street, Dundee DD1 5EH, U.K.; †Department of Physiology, Radboud university medical center, 6500 HB Nijmegen, The Netherlands

**Keywords:** dimerization motif, hypomagnesaemia, ion channel, phosphorylation, protein kinase, transient receptor potential melastatin (TRPM), E, embryonic day, GAPDH, glyceraldehyde-3-phosphate dehydrogenase, HEK, human embryonic kidney, HRP, horseradish peroxidase, HSH, hypomagnesaemia with secondary hypocalcaemia, LDS, lithium dodecyl sulfate, MBP, myelin basic protein, TBST, TBS containing Tween 20, TRPM, transient receptor potential melastatin

## Abstract

Mutations in the gene that encodes the atypical channel-kinase TRPM6 (transient receptor potential melastatin 6) cause HSH (hypomagnesaemia with secondary hypocalcaemia), a disorder characterized by defective intestinal Mg^2+^ transport and impaired renal Mg^2+^ reabsorption. TRPM6, together with its homologue TRPM7, are unique proteins as they combine an ion channel domain with a C-terminally fused protein kinase domain. How TRPM6 channel and kinase activity are linked is unknown. Previous structural analysis revealed that TRPM7 possesses a non-catalytic dimerization motif preceding the kinase domain. This interacts with a dimerization pocket lying within the kinase domain. In the present study, we provide evidence that the dimerization motif in TRPM6 plays a critical role in regulating kinase activity as well as ion channel activity. We identify mutations within the TRPM6 dimerization motif (Leu^1718^ and Leu^1721^) or dimerization pocket (L1743A, Q1832K, A1836N, L1840A and L1919Q) that abolish dimerization and establish that these mutations inhibit protein kinase activity. We also demonstrate that kinase activity of a dimerization motif mutant can be restored by addition of a peptide encompassing the dimerization motif. Moreover, we observe that mutations that disrupt the dimerization motif and dimerization pocket interaction greatly diminish TRPM6 ion channel activity, in a manner that is independent of kinase activity. Finally, we analyse the impact on kinase activity of ten disease-causing missense mutations that lie outwith the protein kinase domain of TRPM6. This revealed that one mutation lying nearby the dimerization motif (S1754N), found previously to inhibit channel activity, abolished kinase activity. These results provide the first evidence that there is structural co-ordination between channel and kinase activity, which is mediated by the dimerization motif and pocket interaction. We discuss that modulation of this interaction could comprise a major regulatory mechanism by which TRPM6 function is controlled.

## Abstract 2

We show that TRPM6 kinase activity is linked to channel activity. This occurs through a kinase-independent mechanism involving the dimerization motif binding to a pocket within the kinase domain. A disease-causing mutation (S1754N) lying nearby the dimerization pocket inactivates kinase activity.

## INTRODUCTION

TRPM6 (transient receptor potential melastatin 6) is an exceptional enzyme possessing a Mg^2+^-permeant ion channel domain and a C-terminal protein kinase moiety [1–4]. Autosomal recessive mutations in the *TRPM6* gene cause HSH (hypomagnesaemia with secondary hypocalcaemia), a serious disorder that affects Mg^2+^ (re)absorption, resulting in decreased serum Mg^2+^ levels [5,6]. This often manifests in convulsions and spasms in early infancy, and can lead to tetany, seizures and cardiac arrhythmias [7]. TRPM6 is specifically expressed in the distal convoluted tubule of the kidney where it plays a prominent role in maintaining blood Mg^2+^ levels (0.7–1.1 mM) [4,8,9]. Furthermore, the role of TRPM6 is emphasized by studies that demonstrated embryonic lethality at mid-gestation (E12.5, where E is embryonic day) in TRPM6-knockout mice due to inability to control Mg^2+^ levels [10,11]. In contrast, its homologue TRPM7 is ubiquitously expressed in all cells and has been proposed to play a general role in controlling cellular growth, survival and proliferation [12–14], as well as cellular Mg^2+^ homoeostasis [15]. Consistent with this, knockout mutations in TRPM7 results in early embryonic lethality before E7 [16]. To date, no mutations in TRPM7 have been linked to human disease.

A critical question is how are the ion channel and catalytic kinase domains regulated and whether the kinase domain is responsible for controlling ion channel activity. Results to date have failed to reveal a major functional link between the channel and kinase activity as truncation of the kinase domain or kinase-inactivating mutations in TRPM6 or TRPM7 do not disrupt ion channel activity [17,18]. However, further more detailed investigation has revealed that kinase truncations/inactivating mutations may have a role in modulating ion channel gating by Mg^2+^ ions, although the mechanism by which this occurs is unclear [15,19–21]. The kinases of TRPM6 and TRPM7 share a sequence homology of nearly 75%. Interestingly, the crystal structure of the TRPM7 kinase domain has been resolved, and the kinase domain was shown to assemble into a homodimer [22]. This dimerization was mediated through a ~30-residue α-helical domain (which we term the ‘dimerization motif’) located N-terminal to the kinase domain that binds to a region termed the ‘dimerization pocket’ on the adjacent kinase domain [22,23]. These regions are situated on the intracellular C-terminal tail of the protein. Mutation or truncation of the dimerization motif in TRPM7 inhibits kinase activity [23], but how this affects ion channel activity has not been studied. In the present study, we have analysed in more detail the role that the TRPM6 dimerization motif plays in controlling kinase and channel activities. We find that mutations in either the dimerization motif or dimerization pocket ablate interaction and kinase activity. Moreover, we demonstrate that these mutations markedly impair ion channel activity. We also show that the protein kinase activity of a dimerization motif mutant can be restored by addition of a dimerization motif peptide. Interestingly, catalytic screening of previously described TRPM6 disease mutants led to the identification of one missense mutation that results in kinase inactivation. Altogether, these findings present new understanding of the interrelationship between the TRPM6 kinase and channel domains. We reveal how the kinase and channel are co-ordinately activated and we discuss the possibility that conformational change in the dimerization motif–dimerization pocket interface plays a critical role in regulating TRPM6 function.

## MATERIALS AND METHODS

### Materials

Glutathione–Sepharose was from GE Healthcare. [γ^32^P]ATP was from PerkinElmer. Anti-FLAG M2–agarose, Tween 20, Colloidal Coomassie Blue staining kit and pre-cast SDS/polyacrylamide Bis-Tris gels were from Invitrogen. CHAPS was from Calbiochem. Triton X-100 was from Sigma. Ampicillin was from Merck. All peptides were synthesized by GL Biochem (Shanghai, China).

### General methods

Restriction enzyme digestions, DNA ligations and other recombinant DNA procedures were performed using standard protocols. All mutagenesis was performed using the QuikChange® site-directed mutagenesis method (Stratagene) with KOD polymerase (Novagen). All DNA constructs were verified by DNA sequencing, which was performed by The Sequencing Service, School of Life Sciences, University of Dundee, using DYEnamic ET terminator chemistry (GE Healthcare) on Applied Biosystems automated DNA sequencers. DNA for mammalian cell transfection was amplified in *Escherichia coli* DH5α strain and plasmid preparation was carried out using Qiagen Maxiprep Kit according to the manufacturer's protocol.

### Buffers

Lysis buffer for mammalian cell lysis contained 50 mM Tris/HCl (pH 7.5), 150 mM NaCl, 1 mM EDTA, 1 mM EGTA, 3% (w/v) CHAPS, 1 mM sodium orthovanadate, 10 mM sodium 2-glycerophosphate, 50 mM sodium fluoride, 10 mM sodium pyrophosphate, 0.27 M sucrose, 0.1% 2-mercaptoethanol, 1 mM benzamidine and 0.1 mM PMSF. Lysis buffer for *E. coli* protein purification contained 50 mM Tris/HCl (pH 7.5), 150 mM NaCl, 1 mM EDTA, 1 mM EGTA, 1% (v/v) Triton X-100, 1 mM sodium orthovanadate, 10 mM sodium 2-glycerophosphate, 50 mM sodium fluoride, 10 mM sodium pyrophosphate, 0.27 M sucrose, 0.1% 2-mercaptoethanol, 1 mM benzamidine and 0.1 mM PMSF. Buffer A contained 50 mM Tris/HCl (pH 7.5) and 0.1 mM EGTA. TBST (TBS containing Tween 20) consisted of Tris/HCl (pH 7.5), 0.15 M NaCl and 0.2% Tween 20. SDS sample buffer was 1× NuPAGE LDS (lithium dodecyl sulfate) sample buffer (Invitrogen), containing 1% (v/v) 2-mercaptoethanol. Homogenization buffer for cellular fractionation contained 50 mM Tris/HCl (pH 7.5), 10 mM KCl, 1.5 mM MgCl_2_, 0.1 mM EDTA, 0.5 mM EGTA and 44 mM sucrose. RIPA buffer contained 50 mM Tris/HCl (pH 7.5), 150 mM NaCl, 0.1% SDS, 0.5% sodium deoxycholate, 1% (v/v) Triton X-100, 0.1% 2-mercaptoethanol, 1 mM benzamidine and 0.1 mM PMSF.

### Cell culture and transfections

HEK (human embryonic kidney)-293 cells were cultured on 10-cm-diameter dishes in DMEM (Dulbecco's modified Eagle's medium) supplemented with 10% (v/v) FBS, 2 mM L-glutamine, 100 units/ml penicillin and 0.1 mg/ml streptomycin. For transfection, each dish of adherent cells was transfected with 5–10 μg of plasmid DNA and 20 μl of 1 mg/ml polyethyleneimine (Polysciences) as described previously [24]. The cells were cultured for a further 48 h and lysed in 0.3 ml of ice-cold lysis buffer per dish, lysates were clarified by centrifugation at 26000 ***g*** at 4°C for 15 min, and the supernatants were frozen in 500 μl aliquots in liquid nitrogen and stored at −20°C. Protein concentrations were determined using the Bradford method.

### Antibodies

The anti-(total GST) antibody was raised in sheep and affinity-purified on the appropriate antigen. The anti-FLAG antibody (F1804) was purchased from Sigma–Aldrich and the anti-GAPDH (glyceraldehyde-3-phosphate dehydrogenase) antibody (ab8245) was purchased from Abcam. Secondary antibodies coupled to HRP (horseradish peroxidase) used for immunoblotting were obtained from Pierce. The anti-Na^+^/K^+^-ATPase antibody was purchased from Cell Signaling Technology.

### Expression and purification of TRPM6 truncates in *E. coli*

DNA for bacterial protein expression was transformed into *E. coli* BL21 cells (Stratagene) and 0.5 litre cultures were grown at 37°C in LB broth containing 100 μg/ml ampicillin until the attenuance at 600 nm was 0.8–1.0. IPTG (250 μM) was added and the cells were cultured for a further 4 h at 37°C. Cells were isolated by centrifugation at 3500 ***g*** for 30 min and lysed in 25 ml of ice-cold lysis buffer by sonication. Lysates were clarified by centrifugation at 26000 ***g*** for 30 min at 4°C followed by incubation with 0.5 ml of glutathione–Sepharose for 1 h at 4°C. The resin was washed thoroughly and proteins were eluted in buffer A containing 0.27 M sucrose and 20 mM glutathione.

### Immunoprecipitation

For immunoprecipitation of FLAG and GST, FLAG M2–agarose beads and glutathione–Sepharose were used respectively. Lysates (0.5–5 mg) were incubated with 10–20 μl of antibody–resin conjugate for 2 h at 4°C with gentle agitation, and the immunoprecipitates were washed three times with lysis buffer containing 0.15 M NaCl and then twice with buffer A. Proteins were eluted by resuspending washed immunoprecipitates in 30 μl of SDS sample buffer.

### Immunoblotting

Cell lysates (20 μg), purified proteins or immunoprecipitates in SDS sample buffer were subjected to electrophoresis on a polyacrylamide gel and transferred on to nitrocellulose membranes. The membranes were incubated for 30 min with TBST containing 5% (w/v) non-fat dried skimmed milk powder. The membranes were immunoblotted in the same buffer overnight at 4°C with the indicated primary antibodies. Sheep antibodies were used at a concentration of 1–2 μg/ml, whereas commercial antibodies were diluted 1000–5000-fold. The blots were then washed with TBST and incubated for 1 h at room temperature (20°C) with HRP-conjugated secondary antibodies diluted 2500-fold in 5% (w/v) non-fat dried skimmed milk in TBST. After repeated washes, the signal was detected with the ECL reagent (GE Healthcare) and the X-ray films were processed in a Konica Minolta Medical SRX-101 film processor.

### Kinase assays

In assays using *E. coli*-expressed TRPM6, reactions were set up in a volume of 20 μl, with substrate MBP (myelin basic protein) at 1 μg and kinase at 0.1–10 μg in buffer A containing 0.1% 2-mercaptoethanol, 10 mM MgCl_2_ and 0.1 mM [γ-^32^P]ATP (~1000 cpm·pmol^−1^). Assays were carried out for 30 min at 30°C with shaking at 1000 rev./min and terminated by addition of SDS sample buffer. In mammalian HEK-293 immunoprecipitation kinase assays, FLAG-tagged or GST-tagged wild-type and mutant TRPM6 was immunoprecipitated from 1–3 mg of cell lysate followed by addition of a reaction volume of 20 μl consisting of buffer containing 0.1% 2-mercaptoethanol, 10 mM MgCl_2_ and 0.1 mM [γ-^32^P]ATP (~1000 cpm·pmol^−1^), with 1 μg of MBP as substrate. Assays were carried out for 30 min at 30°C with shaking at 100 ***g*** and terminated by the addition of SDS sample buffer. For all assays, reaction mixtures were resolved by SDS/PAGE. Proteins were detected by Coomassie Blue staining and gels were imaged using an Epson scanner and dried completely using a gel dryer (Bio-Rad Laboratories). Incorporation of [γ-^32^P]ATP into substrates was analysed by autoradiography using Hyperfilm (GE Healthcare) or quantified by Cerenkov counting.

### Peptide pull-down assays

The peptides biotin-C6-LKSPQEPHHHYSAIERNNLMRLSQ-TIPFTPVQLFAGEEITV (wild-type) and biotin-C6-LKSPQE-PHHHYSAIERNNAMRASQTIPFTPVQLFAGEEITV (mutant) were purchased from GL Biochem. For a peptide pull-down, 1–5 mg of lysate was incubated with 3 μg of the respective peptide for 10 min at 4°C under gentle agitation, followed by 5 min of incubation with 10–20 μl of streptavidin beads at 4°C. The immunoprecipitates were washed three times with lysis buffer containing 0.15 M NaCl and then twice with buffer A. Proteins were eluted by resuspending washed immunoprecipitates in 30 μl of SDS sample buffer.

### PheraStar fluorescence polarization assay

The peptides used for the assay contained a C-terminal linker leading to the peptides LKSPQEPHHHYSAIERNNLMRLSQTIPFTPVQLFAGEEITVGGCCPGCC (wild-type) and LKSPQEPHHHYSAIERNNAMRASQTIPFTPVQLFAGEEITVGGCCPGCC (mutant). The peptides were labelled with Lumio Green (Invitrogen) following the manufacturer's protocol. Fluorescence polarization measurements were performed at room temperature in buffer containing 25 mM Tris/HCl (pH 7.5), 200 mM NaCl and 5 mM 2-mercaptoethanol. Binding assays were performed by combining 100 nM labelled peptide with increasing concentrations of TRPM6 kinase, in a total volume of 30 μl of buffer, and end-point polarization measurements were made using the BMG PheraStar plate reader. The polarization values were measured at an excitation wavelength of 485 nm and an emission wavelength of 538 nm, and were corrected for fluorescent probe alone. At least ten data points were measured for each curve, and each data point was measured in duplicate. Data were analysed using GraphPad Prism 5 and curve-fitting of the data from two independent experiments was performed with one-site specific binding with Hill slope [model Y=*B*_max_X*^h^*/(*K*_d_*^h^*+X*^h^*)] to determine *K*_d_ values.

### Cellular fractionation

HEK-293 cells transfected with FLAG-tagged wild-type and mutant TRPM6 were washed once with PBS and scraped into PBS. The cell lysates were centrifuged at 500 ***g*** for 5 min and the pellet was resuspended in homogenization buffer. Cells were lysed by passing through a tight-fitting Dounce homogenizer 50 times followed by centrifugation at 4000 ***g*** for 10 min. The supernatant was centrifuged at 100000 ***g*** for 1 h in a Beckman type 90Ti rotor. The pellet was resuspended in RIPA buffer and passed five times through a 25-gauge needle. The supernatant was concentrated using an ultraspin column with a membrane with a molecular mass cut-off of 10 kDa. Membrane and cytoplasmic fractions were prepared for immunoblots of FLAG and marker proteins.

### Electrophysiology

Electrophysiological recordings were performed as described previously [4]. Briefly, whole-cell currents were determined in whole-cell configuration using an EPC-9 patch-clamp amplifier controlled by the Patchmaster software (HEKA). Cells were kept in an extracellular bath solution (150 mM NaCl, 10 mM Hepes/NaOH and 1 mM CaCl_2_, pH 7.4). Electrode resistances were between 2 and 3 MΩ when filled with the pipette solution (150 mM NaCl, 10 mM sodium EDTA and 10 mM Hepes/NaOH, pH 7.2). A linear 500 ms voltage ramp from −100 to +100 mV was applied every 2 s from a holding potential of 0 mV. Current densities were determined by normalizing the current amplitude obtained at +80 mV 200 s after break in to the cell membrane capacitance. All experiments were performed at room temperature.

### Mapping of phosphorylation site in the dimerization peptide

A kinase reaction was set up in a volume of 20 μl, with the wild-type biotinylated dimerization peptide and the active (residues 1700–end) kinase fragment in buffer A containing 0.1% 2-mercaptoethanol, 10 mM MgCl_2_ and 0.1 mM [γ-^32^P]ATP (~1000 cpm·pmol^−1^). Assays were carried out for 30 min at 30°C with shaking at 100 ***g*** The reaction was terminated by the addition of LDS sample buffer. DTT was added to a final concentration of 10 mM, the samples were boiled, then alkylated with 50 mM iodoacetamide and subsequently resolved by a Bis-Tris 4–12% polyacrylamide gel, which was stained with Colloidal Coomassie Blue. The phosphorylated dimerization peptide band was excised, cut into smaller pieces and washed sequentially for 10 min on a vibrating platform with 1 ml of each of the following: water, a 1:1 (v/v) mixture of water and acetonitrile, 0.1 M ammonium bicarbonate, a 1:1 mixture of 0.1 M ammonium bicarbonate and acetonitrile, and finally acetonitrile. The gel pieces were dried before incubation at 30°C for 16 h in 25 mM triethylammonium bicarbonate containing 5 g/ml trypsin as described previously [24a]. Following tryptic digestion, more than 95% of the ^32^P radioactivity incorporated in the gel bands was recovered, and the samples were chromatographed on a Vydac 218TP5215 C_18_ column (Separations Group) equilibrated in 0.1% trifluoroacetic acid in water. The column was developed with a linear acetonitrile gradient at a flow rate of 0.2 ml/min and fractions of 0.1 ml were collected and analysed for ^32^P radioactivity by Cerenkov counting. Isolated phosphopeptides were analysed by MS analysis as follows. LC–MS/MS was performed using a linear ion trap–orbitrap hybrid mass spectrometer (OrbiTrap Classic) equipped with a nanoelectrospray ion source (Proxeon Biosystems or Thermo) and coupled to a Proxeon EASY-nLC system. Peptides were typically injected on to a Thermo/Dionex Acclaim PepMap100 reverse-phase C_18_ column, 75 m×15 cm, with a flow rate of 300 nl/min and eluted with a 40 min linear gradient of 95% solvent A (2% acetonitrile and 0.1% formic acid in water) to 35% solvent B (90% acetonitrile and 0.08% formic acid in water). The instrument was operated with the ‘lock mass’ option to improve the mass accuracy of precursor ions, and data were acquired in the data-dependent mode, automatically switching between MS and MS/MS acquisition. Full-scan spectra (*m*/*z* 340–1800) were acquired in the orbitrap with resolution *R*=60000 at *m*/*z* 400 (after accumulation to a target value of 1000000). The five most intense ions, above a specified minimum signal threshold, based on a low-resolution (*R*=20000) preview of the survey scan, were fragmented by collision-induced dissociation and recorded in the linear ion trap (target value of 5000). Multistage activation was used to provide an MS^3^ scan of parent ions showing a neutral loss of 48.9885, 32.6570 and 24.4942, allowing for 2^+^, 3^+^ and 4^+^ ions respectively. The resulting MS^3^ scan was automatically combined with the relevant MS/MS scan before data analysis using the Mascot search algorithm (http://www.matrixscience.com) run on a local server.

### ^32^P-labelled phosphopeptide sequence analysis

The site of phosphorylation of the ^32^P-labelled peptides was determined by solid-phase Edman degradation, on an Applied Biosystems 494C sequencer, of the peptide coupled to Sequelon-AA membrane (Applied Biosystems) as described previously [25].

## RESULTS

### The TRPM6 dimerization domain is critical for kinase activity

**Figure 1:**
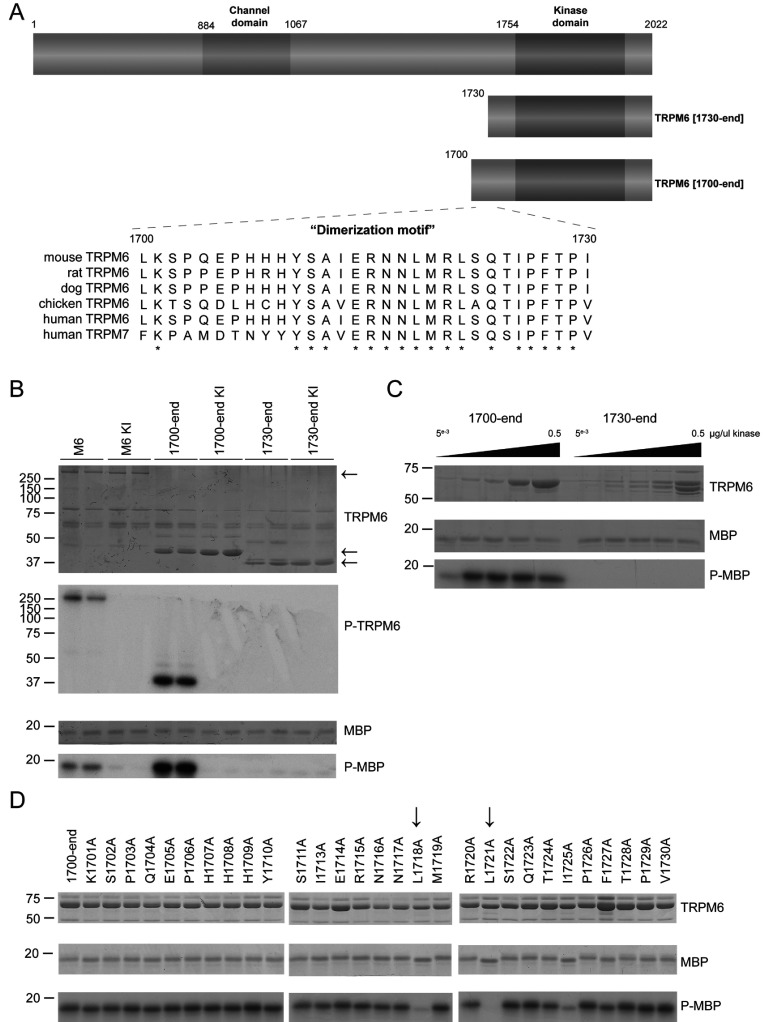
Characterization of TRPM6 (auto)phosphorylation in C-terminal truncation mutants (**A**) Schematic representation of the domain structure of TRPM6 demonstrating the ion channel domain and the α-kinase domain. The lower panel demonstrates a multiple sequence alignment of the 1700–1730 region of TRPM6 among different species along with human TRPM7. Asterisks represent conserved amino acids. (**B**) The Coomassie Blue-stained SDS gel indicates the FLAG-tagged full-length and truncated forms of wild-type and kinase-inactive (KI) TRPM6. Phosphorylation of MBP and TRPM6 itself was determined by autoradiography (lower panels). (**C**) Increasing amounts of GST-purified kinase fragments TRPM6-(1700–end) and TRPM6-(1730–end) were subjected to a kinase assay using MBP as substrate. Proteins were separated by SDS/PAGE, stained with Coomassie Blue (top and middle panels) and phosphorylation was detected by autoradiography (bottom panel). (**D**) Alanine-scanning mutagenesis has been performed on the 1700–1730 region. The indicated GST-tagged mutants were immunoprecipitated from overexpressed HEK-293 cells, and kinase activity was assayed using MBP as substrate. Proteins were separated by SDS/PAGE, stained with Coomassie Blue (top and middle panels) and phosphorylation was detected by autoradiography (bottom panel). Molecular masses are indicated in kDa.

Previous studies have focused on monitoring TRPM7 kinase activity by measuring its autophosphorylation and phosphorylation of the exogenous substrate MBP [2,3,26]. To analyse the TRPM6 kinase activity, we first expressed in HEK-293 cells full-length wild-type TRPM6 as well as a catalytically inactive TRPM6 mutant in which the kinase catalytic lysine residue is replaced by arginine (K1804R), which has been reported previously to abolish autophosphorylation [17]. Immunoprecipitation of the full-length protein confirmed that TRPM6 undergoes autophosphorylation and is able to phosphorylate MBP in a manner that is ablated by the kinase-inactivating mutation (Figure 1B).

Structural analysis of the kinase domain of TRPM7 revealed that it contained a non-catalytic N-terminal extension encompassing a ~30-residue α-helical region, which mediates dimerization and we therefore termed this the ‘dimerization motif’ [22]. The dimerization motif is highly conserved in TRPM6 and corresponds to residues 1700–1730 in human TRPM6 (Figure 1A). To explore the role of the dimerization motif in TRPM6, we expressed in HEK-293 cells fragments that encompass [TRPM6-(1700–end)] or lack [TRPM6-(1730–end)] this motif. This revealed that TRPM6-(1700–end) containing the dimerization motif displayed significant catalytic MBP as well as autophosphorylation activity that is abolished by the K1804R mutation (Figure 1B). In contrast, TRPM6-(1730–end) lacking the dimerization motif displayed no MPB kinase or autophosphorylation activity (Figure 1B). We also expressed these catalytic fragments of TRPM6 in *E. coli* and observed that TRPM6-(1700–end) was active and TRPM6-(1730–end) displayed no activity, even at high concentrations (Figure 1C).

To establish which residues in the dimerization motif were critical for kinase activity, we undertook an alanine scan of each of the amino acids in the 1700–1730 region and delineated how individual mutations affected kinase activity of TRPM6-(1700–end). This revealed that mutation of two highly conserved leucine residues (Leu^1718^ and Leu^1721^) led to a significant loss in kinase activity, whereas the other mutations had little or no effect (Figure 1D).

### The dimerization domain directly binds to and activates TRPM6 kinase

**Figure 2:**
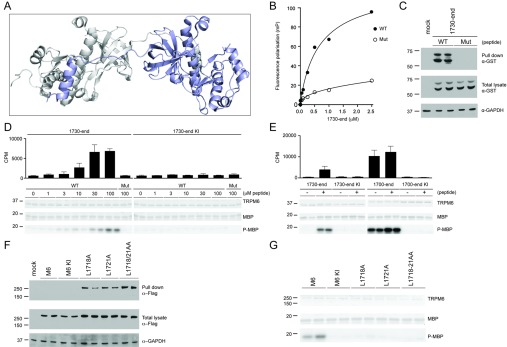
Identification of dimerization motif residues in TRPM6 (**A**) Structural model of TRPM7 α-kinase depicting the N-terminal non-catalytic α-helix of one monomer that interacts with the kinase segment of the other monomer. (**B**) Fluorescence polarization analysis of wild-type (WT) and mutant (Mut) dimerization motif peptides with *E. coli*-purified GST-tagged TRPM6-(1730–end). (**C**) GST-tagged TRPM6-(1730–end) was expressed in HEK-293 cells, and lysates were subjected to a peptide pull-down, as described in the Materials and methods section, using either wild-type (WT) or mutant (Mut) dimerization motif peptide. The samples were analysed by immunoblotting with anti-GST antibody (top panel). Total cell extracts were immunoblotted with GST antibody (middle panel), using GAPDH expression as loading control (bottom panel). (**D**) Immunoprecipitation of FLAG-tagged TRPM6-(1730–end) wild-type and kinase-inactive TRPM6 from HEK-293 cell lysate was followed by a kinase assay using increasing concentrations of the wild-type (WT) dimerization motif peptide, and highest concentrations (100 μM) for mutant (Mut) peptide. Proteins were separated by SDS/PAGE, stained with Coomassie Blue (top and middle gels) and MBP phosphorylation was detected by autoradiography (bottom gel). Incorporated radioactive counts are depicted as a histogram. (**E**) FLAG-tagged TRPM6-(1730–end) and TRPM6-(1700–end), both wild-type and kinase-inactive (KI) were immunoprecipitated and subjected to a kinase assay without (−) or with (+) 30 μM dimerization motif peptide. Proteins were separated by SDS/PAGE, stained with Coomassie Blue (top and middle gels) and MBP phosphorylation was detected by autoradiography (bottom gel). Incorporated radioactive counts are depicted as a histogram. (**F**) Full-length FLAG-tagged TRPM6 wild-type, kinase-inactive (KI) and indicated mutants from HEK-293 cells lysate were subjected to a peptide pull-down, as described in the Materials and methods section, using the wild-type dimerization motif peptide. The pull-down samples were analysed by immunoblotting with anti-FLAG antibody (top panel). Total cell extracts were immunoblotted with anti-FLAG antibody (middle panel), using GAPDH expression as loading control (bottom panel). (**G**) Immunoprecipitated FLAG-tagged TRPM6 and indicated mutants were subjected to a kinase assay using MBP as substrate. Proteins were separated by SDS/PAGE, stained with Coomassie Blue (top and middle panels) and MBP phosphorylation was detected by autoradiography (bottom panel). Molecular masses are indicated in kDa.

Closer inspection of the TRPM7 dimeric kinase domain structure revealed that the dimerization motif forms an extended α-helix which undergoes extensive interactions with a region in the kinase domain of the other monomer termed the ‘dimerization pocket’ [22] (Figure 2A). We next utilized fluorescence polarization technology, a sensitive method to evaluate interactions between proteins and peptides [27]. Using this methodology, we first evaluated how polarization of a fluorescently labelled peptide encompassing the dimerization motif was affected by titration of TRPM6-(1730–end) (which lacks the dimerization motif). This revealed a dose-dependent increase in fluorescence polarization following addition of increasing concentrations of TRPM6-(1730–end) (Figure 2B), which indicates binding. Kinetic analysis of the data demonstrated that interaction data could be fitted using a variable slope model to a one-site-binding equation (Hill slope of 1.0), with *K*_d_ calculated as 0.7 μM (*K*_d_ 0.713 μM). In contrast, when analysis was undertaken with a mutant dimerization motif peptide containing the two leucine mutations (L1718A+L1721A) that impaired TRPM6 catalytic activity (Figure 1D), only background increases in fluorescence polarization were observed (Figure 2B). Next, we addressed whether it was possible to affinity-purify TRPM6-(1730–end) from HEK-293 cell extracts by employing a biotinylated dimerization motif peptide conjugated to streptavidin–Sepharose. We found that this peptide, but not the mutant (L1718A+L1721A), could efficiently purify TRPM6-(1730–end) (Figure 2C).

We next investigated whether this binding of the dimerization motif peptide could restore catalytic activity of the inactive TRPM6-(1730–end) fragment. Addition of the dimerization motif peptide resulted in a dose-dependent activation of TRPM6-(1730–end) with half-maximal activation observed at ~15 μM peptide (Figure 2D). In contrast, concentrations of as high as 100 μM mutant (L1718A+L1721A) peptide failed to significantly activate TRPM6-(1730–end). We observed that the specific activity of TRPM6-(1730–end) incubated with 30 μM peptide corresponded to approximately half of the specific kinase activity of the TRPM6-(1700–end) fragment containing the dimerization motif (Figure 2E). Addition of the dimerization motif peptide did not enhance the kinase activity of TRPM6-(1700–end) further (Figure 2E).

Our data suggest that the TRPM6 dimerization motif binds and activates the kinase domain with Leu^1718^ and Leu^1721^ playing a critical role. If this model is correct, we would predict that the full-length tetrameric TRPM6 protein functions through dimerization, and thereby each monomeric dimerization motif would occupy the dimerization pocket of its adjacent kinase domain, leaving no scope to bind the dimerization motif peptide *in trans*. Consistent with this, we find that full-length wild-type and kinase-inactive TRPM6 expressed in HEK-293 cells do not interact with the biotinylated dimerization motif peptide conjugated to streptavidin–Sepharose (Figure 2F). In contrast, full-length TRPM6 mutants in which the dimerization pocket would be unoccupied as a result of L1718A+L1721A mutations is rendered capable of interacting with the biotinylated dimerization motif peptide conjugated to streptavidin–Sepharose (Figure 2F). We also demonstrated that individual or double L1718A and L1721A mutations ablated kinase activity in full-length TRPM6, confirming the importance of this dimerization motif interaction in regulating kinase activity (Figure 2G).

### Identification of critical residues in the TRPM6 dimerization pocket

**Figure 3:**
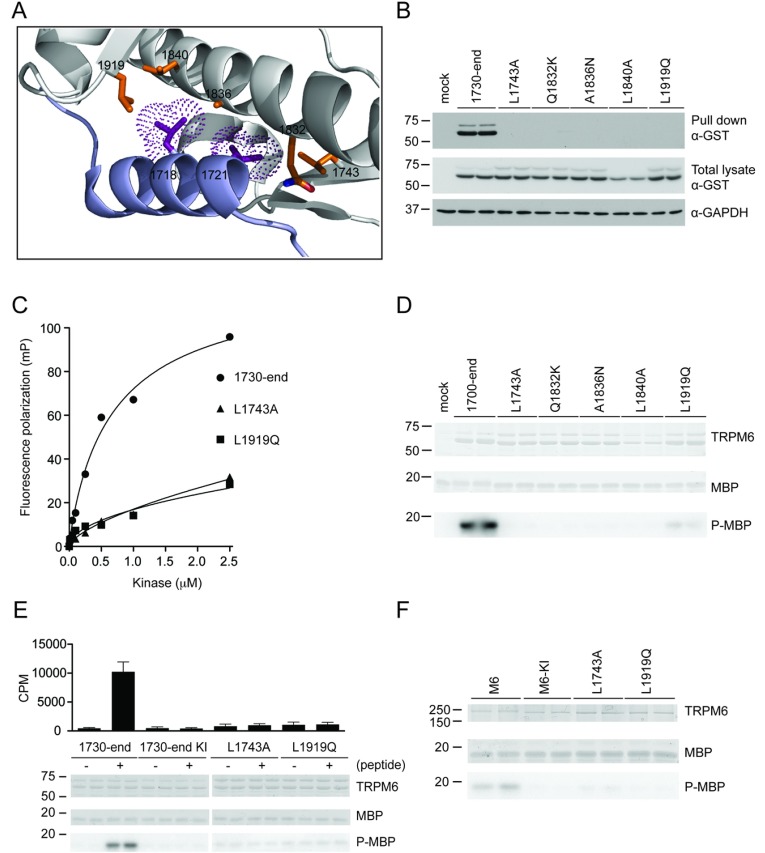
Analysis of the dimerization pocket residues (**A**) Structural model of TRPM6 based on TRPM7 α-kinase depicting the binding of Leu^1718^ and Leu^1721^ to other residues in the α-kinase domain. (**B**) GST-tagged TRPM6-(1730–end) and the indicated mutants were expressed in HEK-293 cells. Subsequently, cell lysate was subjected to a peptide pull-down, as described in the Materials and methods section, using wild-type dimerization motif peptide. The samples were analysed by immunoblotting with anti-GST antibody (top panel). Total cell extracts were immunoblotted with anti-GST antibody (middle panel), using GAPDH expression as loading control (bottom panel). (**C**) Fluorescence polarization analysis of the dimerization motif peptide with *E. coli* purified GST-tagged TRPM6-(1730–end) and indicated mutants. (**D**) Immunoprecipitation of GST-tagged TRPM6-(1700–end) and indicated mutants from HEK-293 cell lysate was followed by a kinase assay. Proteins were separated by SDS/PAGE, stained with Coomassie Blue (top and middle panels) and MBP phosphorylation was detected by autoradiography (bottom panel). (**E**) GST-tagged TRPM6-(1730–end), both wild-type and kinase-inactive (KI), and L1743A and L1919Q mutants were expressed in HEK-293 cells, followed by immunoprecipitation and then subjected to a kinase assay without (−) or with (+) 30 μM dimerization motif peptide. Proteins were separated by SDS/PAGE, stained with Coomassie Blue (top and middle gels) and MBP phosphorylation was detected by autoradiography (bottom gel). Incorporated radioactive counts are depicted as a histogram. (**F**) Immunoprecipitated FLAG-tagged TRPM6 and the indicated mutants were subjected to a kinase assay using MBP as substrate. Proteins were separated by SDS/PAGE, stained with Coomassie Blue (top and middle panels) and MBP phosphorylation was detected by autoradiography (bottom panel). Molecular masses are indicated in kDa.

The critical dimerization motif residues Leu^1718^ and Leu^1721^ are conserved in TRPM7 (Figure 1A), where the equivalent Leu^1561^ and Leu^1564^ make several hydrophobic interactions with four kinase domain residues (Gln^1674^, Ala^1678^, Leu^1681^ and Leu^1761^) and a non-catalytic N-terminal residue (Leu^1585^) within the dimerization pocket (Figure 3A). Interestingly, the equivalent four catalytic residues (Gln^1832^, Ala^1836^, Leu^1840^ and Leu^1919^) and the non-catalytic residue (Leu^1743^) are conserved in TRPM6 (Supplementary Fig-ure S1 at http://www.biochemj.org/bj/460/bj4600165add.htm). To investigate the role that these residues play in enabling binding of the dimerization motif, the TRPM6-(1730–end) wild-type and dimerization pocket mutants (L1743A, Q1832K, A1836N, L1840A and L1919Q) were expressed in HEK-293 cells and tested for their ability to bind to the biotinylated dimerization motif peptide conjugated to streptavidin–Sepharose. This revealed that individual mutation of all of these residues abolished binding to the peptide (Figure 3B). Fluorescence polarization binding assays confirmed that two of these mutations (L1743A and L1919Q) inhibited interaction with the fluorescently labelled dimerization motif peptide (Figure 3C). Consistent with occupancy of the dimerization pocket being required for kinase activity, we found that incorporating the dimerization pocket mutations in either TRPM6-(1700–end) (Figure 3D) or full-length TRPM6 (Figure 3F) abolished kinase activity. Finally, we observed that the ability of the dimerization peptide to activate TRPM6-(1730–end) was prevented by the L1743A and L1919Q dimerization pocket mutations (Figure 3E).

### Importance of dimerization binding for TRPM6 channel activity

**Figure 4:**
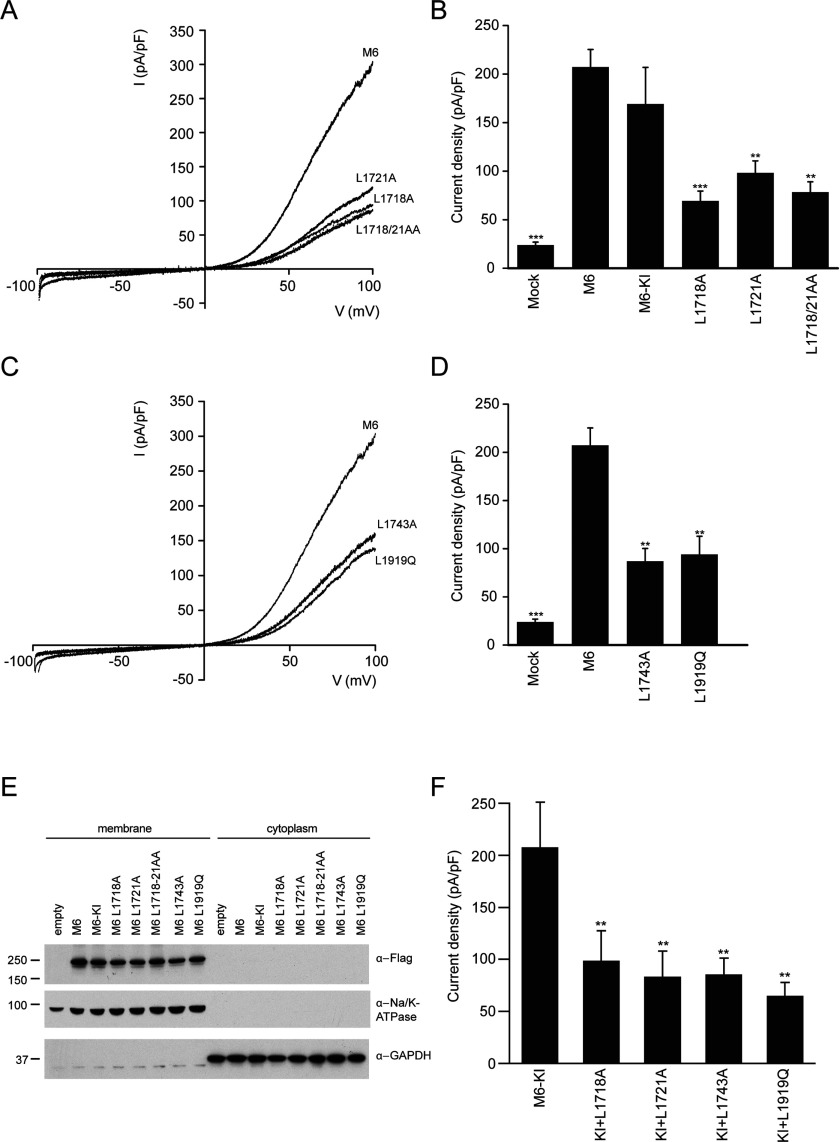
Dimerization interactions are linked to the channel function (**A**) Representative current recorded after 200 s of stimulation by a voltage ramp between −100 and +100 mV of HEK-293 cells transfected with wild-type and kinase-inactive (KI) TRPM6 and the indicated mutants (L1718A, L1721A and L1718/21AA). (**B**) Histogram summarizing the current density (pA/pF) at +80 mV of TRPM6 and indicated mutants. **P<0.05 and ***P<0.01 compared with wild-type TRPM6 (*n*=11–50 cells). (**C**) Representative current recorded after 200 s of stimulation by a voltage ramp between −100 and +100 mV of HEK-293 cells transfected with TRPM6 and the indicated mutants (L1743A and L1919Q). (**D**) Histogram summarizing the current density (pA/pF) at +80 mV of TRPM6 and the indicated mutants. **P<0.05 and ***P<0.01 compared with wild-type TRPM6 (*n*=11–50 cells). (**E**) Membrane and cytoplasmic fractions were obtained by ultracentrifugation to determine the expression of FLAG-tagged TRPM6 and all mutants. Lysates were immunoblotted with anti-FLAG antibody, and Na^+^/K^+^-ATPase and GAPDHs used as membrane and cytosolic marker were respectively. Molecular masses are indicated in kDa. (**F**) Histogram summarizing the current density (pA/pF) at +80 mV of kinase-inactive (KI) TRPM6 and indicated double mutants (KI+L1718A, KI+L1721A, KI+L1743A and KI+L1919Q). **P<0.05 compared with TRPM6 KI (*n*=13–25 cells). Results are means ± S.E.M.

Next, we examined whether binding of the dimerization motif to the dimerization pocket in the kinase domain plays a role in regulating TRPM6 channel function. To address this question, full-length wild-type TRPM6, and kinase-inactive (K1804R), dimerization motif (L1718A, L1721A and L1718A+L1721A) and dimerization pocket (L1743A and L1919Q) mutants were expressed in HEK-293 cells and we studied channel activity by whole-cell patch-clamp recordings. TRPM6 is a constitutively active cation channel that is tightly regulated by intracellular Mg^2+^ and Mg^2+^-ATP. Therefore TRPM6 currents are induced by internal Mg^2+^ depletion using Mg^2+^-free pipette solutions containing EDTA. TRPM6 channels produce small inward currents at physiological negative membrane potentials and distinct outward rectification at positive membrane potentials (Figures 4A and 4B). Consistent with previous studies, the kinase-inactive TRPM6 has a similar current–voltage relationship (Figure 4A) and amplitude (Figure 4B) as wild-type. Interestingly, however, the dimerization motif mutants displayed significant (~3-fold) diminished current amplitudes (Figures 4A and 4B). We also observed that the current activity of the TRPM6 dimerization pocket mutants was significantly reduced (Figures 4C and 4D). As a control, we demonstrated that the membrane expression of the dimerization motif and dimerization pocket mutants was equal to that of the wild-type and kinase-inactive TRPM6, suggesting that these mutations do not hamper expression and transport to the plasma membrane (Figure 4E). Furthermore, we have demonstrated that mutation of the dimerization motif or pocket residues in kinase-inactive TRPM6 also significantly reduce the current amplitudes. This provides further evidence that the mechanism by which dimerization stimulates TRPM6 ion channel activity is independent of kinase activity (Figure 4F).

### Impact on kinase activity of disease-causing missense mutations

**Figure 5:**
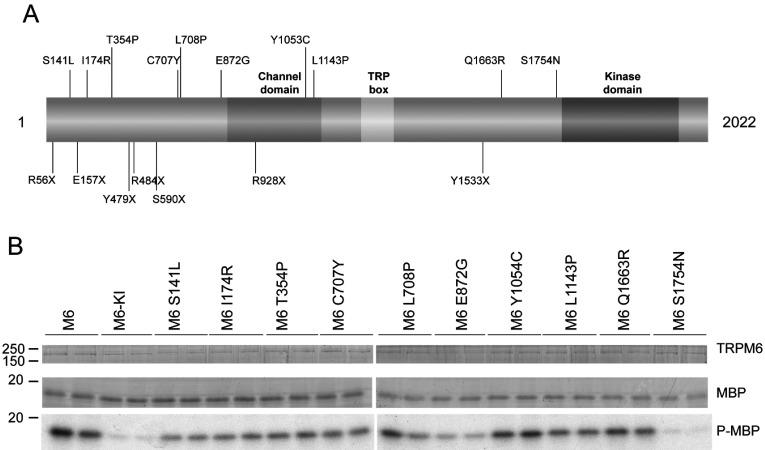
The disease-causing mutation S1754N also abrogates kinase function in TRPM6 (**A**) Schematic representation of the domain structure of TRPM6 depicting the missense mutations identified in patients suffering from HSH. (**B**) Immunoprecipitated FLAG-tagged wild-type and kinase-inactive (KI) TRPM6 and indicated mutants were subjected to a kinase assay using MBP as substrate. Proteins were separated by SDS/PAGE, stained with Coomassie Blue (top and middle panels) and MBP phosphorylation was detected by autoradiography (bottom panel). Molecular masses are indicated in kDa.

In addition to a number of frameshift mutations, ten missense mutations in TRPM6 have been identified that cause HSH [5,6,28]. These are all located outside the catalytic domain (Figure 5A). Interestingly one of the residues, Ser^1754^, lies just before the kinase domain and inspection of the equivalent residue in the TRPM7 structure suggests that is located in close proximity to the dimerization motif–dimerization pocket interaction. To screen the effects that the missense mutations have on TRPM6 activity, we expressed full-length TRPM6 wild-type, kinase-inactive and all ten mutants in HEK-293 cells and assessed kinase activity after immunoprecipitation. All TRPM6 mutants were expressed at equal levels to that of wild-type and displayed similar kinase activity, except for S1754N which was catalytically inactive (Figure 5B). Parallel to the other dimerization pocket mutants analysed in the present study (L1743A and L1919Q, Figure 4D), a recent study has shown that the S1754N mutant also displays markedly reduced channel activity [28].

## DISCUSSION

TRPM6, together with its homologue TRPM7, are unique as they are the only known examples in Nature that consist of both an ion channel and a kinase domain within the same polypeptide chain. Before the present study, the mechanism by which channel and kinase activity were co-ordinated was unknown. The main conclusion of the present study is demonstrating for the first time a direct link between the kinase and channel function of TRPM6. Our data reveal that binding of the dimerization motif to the dimerization pocket is essential for both kinase and ion channel activity. We identify critical residues required for this interaction and demonstrate that mutation of these residues leads to a significant suppression of both kinase and channel activity. Significantly, we also characterized a HSH disease-causing missense mutation, lying within the dimerization pocket–dimerization motif interaction (S1754N), that inhibits kinase (Figure 5C) as well as channel activity [28].

Each TRPM6 subunits comprises six transmembrane segments, an intracellular N-terminal tail (~800 residues) and an intracellular C-terminal segment encompassing the kinase domain (~1000 residues). Previous studies indicated that TRPM6 operates as a tetramer in which the channel pore is built up by the membrane-spanning regions of four TRPM6 subunits [29,30]. The data suggest that in the channel and kinase-active conformation, the dimerization motif of one subunit interacts with a dimerization pocket of another subunit. The present study proposes that all mutations that we have tested that abolish this interaction also impair kinase as well as channel activity. The present paper is the first report of individual mutations that disrupt both catalytic activities of TRPM6 and strongly suggests that channel activity is linked to kinase function.

It should be noted that the physiologically relevant regulation of TRPM6 and TRPM7 channel activity is a controversial topic. Previous studies have demonstrated that the kinase domain is redundant for TRPM6 or TRPM7 channel function, but is involved in the modulation of channel activity [15–18,21]. Intracellular levels of Mg^2+^ or Mg^2+^-ATP are important factors for controlling TRPM6 and TRPM7 activity [4,12,19,21]. A recent study provided support for the TRPM6 kinase domain as a Mg^2+^/Mg^2+^-ATP-sensing mechanism regulating TRPM6/TRPM7 channel activity [21]. Together with our results, this suggests that kinase activity is not essential for the dimerization motif–dimerization pocket interaction, which stimulates channel function. On the contrary, it implies that the dimerization interactions provide the basis for TRPM6 functioning. Advanced ways to study TRPM6 in a more physiological context could provide further answers on the regulatory mechanism for dimerization. Future work towards optimizing Mg^2+^-sensitive probes for TRPM6 channel measurements would greatly improve this.

Interestingly, TRPM7-deficient mice lacking the kinase domain show early embryonic lethality, and embryonic stem cells from these mice exhibit growth inhibition that can be restored by Mg^2+^ supplementation [31], which points towards a physiological importance of the kinase domain. These stem cells produce active TRPM7 channels, albeit with reduced current magnitude [31]. This suggests that the channel adopts an active conformation following truncation of the kinase domain. In future work, it would be interesting to test how a knockin mutation that ablated dimerization motif–dimerization pocket interaction affected channel function. It would also be interesting to explore how a knockin mutation that abolished TRPM6/TRPM7 kinase function affected phenotype and channel activity.

It has been shown previously that TRPM6 can assemble into complexes with TRPM7, at least in co-expression studies [15,32]. Interestingly, it has been shown that TRPM6–TRPM7 complexes displayed different channel characteristics compared with TRPM6 and TRPM7 alone [21,32]. As the sequence of the dimerization motif (Figure 1A) and pocket (Supplementary Figure S1) are highly conserved in TRPM6 and TRPM7, this suggests that the dimerization motif of TRPM6 could interact with the dimerization pocket of TRPM7 and vice versa. It would be interesting to see whether this cross-dimerization contributes to different channel characteristics observed and potentially some of the dimerization motif and dimerization pocket mutants could be utilized to study this.

In future work, it will be critical to explore whether the physiological stimuli that regulate TRPM6 function do so by influencing the dimerization motif–dimerization pocket interaction. Another critical question will be to define what are the physiological substrates of the TRPM6 kinase and how phosphorylation of these proteins modulates downstream biology. To date, the only clear-cut TRPM6 substrates identified are a series of autophosphorylation sites lying between the kinase and channel domains [17,26]. Interestingly, a recent study for *in vivo* TRPM7 phosphorylation sites identified autophosphorylation of two residues (Ser^1565^ and Ser^1567^) that are located within the dimerization motif and are conserved in TRPM6 (Ser^1722^ and Thr^1724^) [33]. We have been able to confirm that Thr^1724^ comprises a major TRPM6 autophosphorylation site (Supplementary Figure S2 at http://www.biochemj.org/bj/460/bj4600165add.htm). An attractive hypothesis is that autophosphorylation of Thr^1724^ and potentially other residues could induce conformational changes that modulate the dimerization motif–dimerization pocket interaction and hence influence channel activity. So far, we have mutated Thr^1724^ as well as a number of other TRPM6 phosphorylation sites that we have mapped (Ser^1281^, Ser^1304^, Ser^1306^, Ser^1329^, Ser^1365^, Ser^1560^, Ser^1562^, Ser^1563^, Ser^1658^, Ser^1685^, Ser^1689^, Ser^1722^, Thr^2011^ and Ser^2015^), but, to date, none of these had a significant impact on kinase activity. We have also synthesized a dimerization motif peptide phosphorylated at Thr^1724^, but observed that this peptide interacted and activated TRPM6-(1730–end) similarly to the non-phosphorylated dimerization motif peptide (Supplementary Figure S2). Further work is required to understand how phosphorylation of TRPM6 influences its activity.

A key question concerns the mechanism by which channel activity is disrupted by inhibiting the interaction of the dimerization motif to the dimerization pocket on an adjacent TRPM6 kinase domain. One possibility would be that the dimerization motif or dimerization pocket could form an inhibitory interaction with the N-terminal tail or channel domains. As both the N-terminal and C-terminal non-transmembrane regions are localized in the intracellular compartment, this offers scope for potential interactions. In future work, it would be interesting to explore whether such interactions can be observed and defining how critical these are in regulating TRPM6. It should be noted that none of the N-terminal or transmembrane-located disease mutants we have analysed that inhibit channel activity [28,34] affect kinase activity (Figure 5B). Furthermore, it would also be of interest to undertake further analysis on the large cytoplasmic N-terminal domain of TRPM6 that possesses no obvious function, which might provide further clues to how TRPM6 is regulated and functions.

## Online data

Supplementary data
