# Supplementary material for: Kinase and channel activity of TRPM6 are co-ordinated by a dimerization motif and pocket interaction
Source: Biochem J. 2014 May 13;460(Pt 2):165–75. doi: 10.1042/BJ20131639 (PMC4019984; doi:10.1042/BJ20131639)
Supplement: Supplementary data [file bj4600165add.pdf]

BJ Signal

Biochem. J. (2014) **460**, 165–175 (Printed in Great Britain) doi:10.1042/BJ20131639

online data

SUPPLEMENTARY ONLINE DATA

Kinase and channel activity of TRPM6 are co-ordinated by a dimerization motif and pocket interaction

Jenny VAN DER WIJST*[1], Maxime G. BLANCHARD†, Helen I. WOODROOF*, Thomas J. MACARTNEY*, Robert GOURLAY*, Joost G. HOENDEROP†, René J. BINDELS† and Dario R. ALESSI*[1]

*MRC Protein Phosphorylation and Ubiquitylation Unit, College of Life Sciences, University of Dundee, Dow Street, Dundee DD1 5EH, U.K.
†Department of Physiology, Radboud university medical center, 6500 HB Nijmegen, The Netherlands

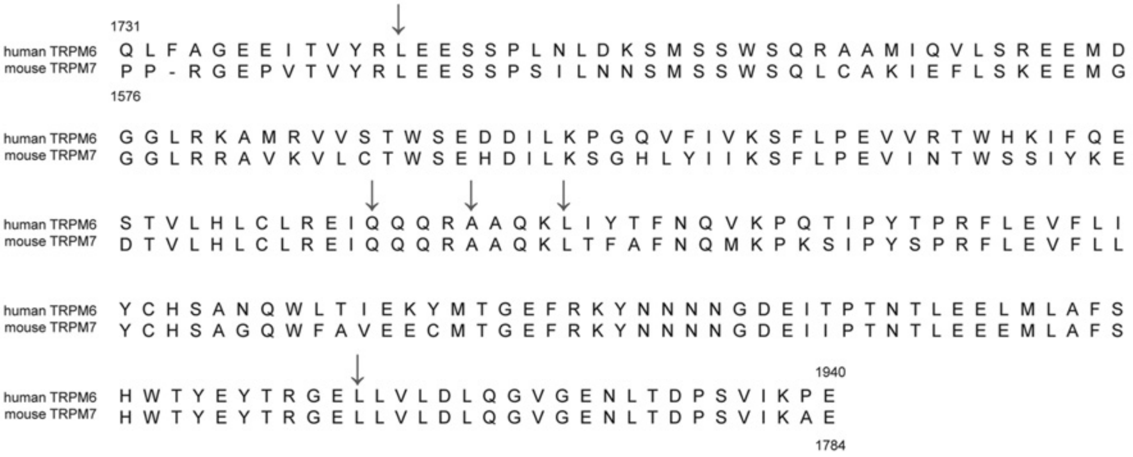

Figure S1 Sequence alignment between human TRPM6 and mouse TRPM7

Conserved dimerization pocket motif residues (Leu^{1743}, Gln^{1832}, Ala^{1836}, Leu^{1840} and Leu^{1919}) are indicated by arrows.

[1] Correspondence may be addressed to either of these authors (email jenny.vanderwijst@radboudumc.nl or d.r.alessi@dundee.ac.uk).

© 2014 The Author(s)
The author(s) has paid for this article to be freely available under the terms of the Creative Commons Attribution Licence (CC-BY) (http://creativecommons.org/licenses/by/3.0/) which permits unrestricted use, distribution and reproduction in any medium, provided the original work is properly cited.

Biochemical Journal www.biochemj.org

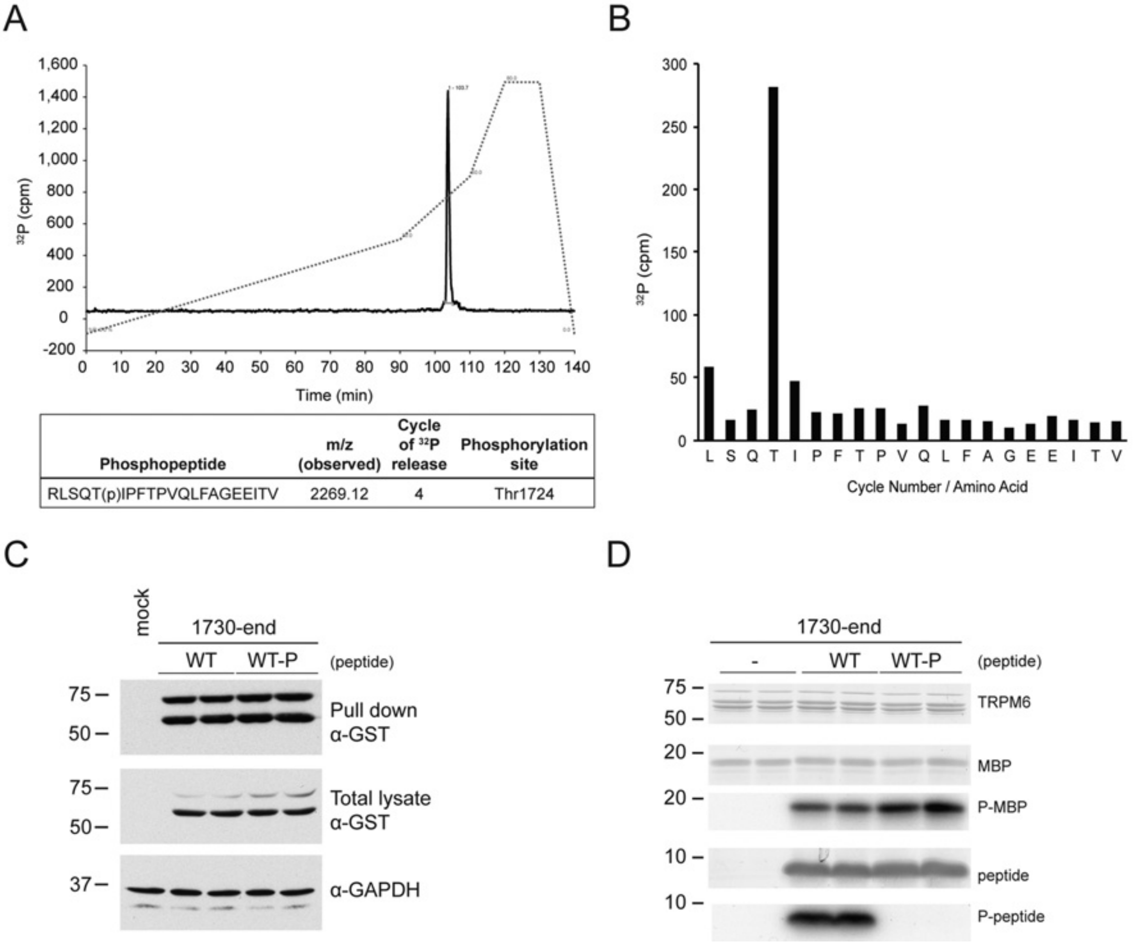

Phosphopeptide	m/z (observed)	Cycle of ^{32}P release	Phosphorylation site
RLSQT(p)IPFTPVQLFAGEEITV	2269.12	4	Thr1724

Figure S2 Identification and characterization of phosphosite Thr[1724] in the dimerization motif peptide

(**A**) Mapping of phosphopeptides on the dimerization motif peptide after phosphorylation by TRPM6 *in vitro*. GST-tagged TRPM6-(1700–end) was immunoprecipitated and subjected to a kinase assay including the biotinylated dimerization peptide (500 μM). Assays were terminated by addition of LDS loading buffer, and proteins were separated by SDS/PAGE. Proteins were detected by Colloidal Coomassie Blue staining, and the stained phosphorylated dimerization motif peptide was digested with trypsin, followed by reverse-phase HPLC on a Vydac C_{18} column equilibrated with 0.1 % trifluoroacetic acid, and the column was developed with an acetonitrile gradient (broken line). The flow rate was 0.2 ml/min and fractions (0.1 ml each) were collected and analysed for ^{32}P radioactivity by Cerenkov counting. A major ^{32}P-labelled peak was identified. (**B**) Solid-phase sequence analysis of the tryptic phosphopeptide RLSQT(p)IPFTPVQLFAGEEITV (residues 19–41). The peptide was phosphorylated at threonine at position 22, corresponding to Thr[1724] in TRPM6. (**C**) GST-tagged TRPM6-(1730–end) was expressed in HEK-293 cells, and lysates were subjected to a peptide pull-down, as described in the Materials and methods section of the main text, using either wild-type (WT) or phospho-wild-type (WT-P) dimerization motif peptide. The samples were analysed by immunoblotting with anti-GST antibody (top panel). Total cell extracts were immunoblotted with anti-GST antibody (middle panel), using GAPDH expression as loading control (bottom panel). (**D**) GST-tagged TRPM6-(1730–end) was expressed in HEK-293 cells, followed by immunoprecipitation and subjected to a kinase assay using 30 μM of either wild-type (WT) or phospho-wild-type (WT-P) dimerization motif peptide. Proteins were separated by SDS/PAGE, stained with Coomassie Blue, and MBP phosphorylation as well as peptide phosphorylation were detected by autoradiography. Molecular masses are indicated in kDa.

Received 13 December 2013/28 February 2014; accepted 21 March 2014
Published as BJ Immediate Publication 21 March 2014, doi:10.1042/BJ20131639

© 2014 The Author(s)
The author(s) has paid for this article to be freely available under the terms of the Creative Commons Attribution Licence (CC-BY) (http://creativecommons.org/licenses/by/3.0/)
which permits unrestricted use, distribution and reproduction in any medium, provided the original work is properly cited.
